# Prevalence and trend of anemia in children with inflammatory bowel disease: A national register‐based cohort study

**DOI:** 10.1002/jpn3.70029

**Published:** 2025-03-31

**Authors:** Giulia D'Arcangelo, Marco Brecciaroli, Giulia Gagliostro, Dalila Auletta, Salvatore Pellegrino, Serena Arrigo, Francesco Graziano, Erasmo Miele, Maria Teresa Illiceto, Patrizia Alvisi, Dario Dilillo, Costantino De Giacomo, Paolo Lionetti, Maria Pastore, Mara Cananzi, Matteo Bramuzzo, Roberto Panceri, Lorenzo Norsa, Marina Aloi, The SIGENP IBD Working Group

**Affiliations:** ^1^ Pediatric Gastroenterology and Liver Unit, Maternal and Child Health Department Sapienza University of Rome Rome Italy; ^2^ Pediatric Gastroenterology and Cystic Fibrosis Unit, Department of Human Pathology in Adulthood and Childhood “G. Barresi” University Hospital “G. Martino” Messina Italy; ^3^ Gastroenterology Unit IRCCS Istituto Giannina Gaslini Genova Italy; ^4^ Pediatric Unit Villa Sofia Cervello Hospital Palermo Italy; ^5^ Department of Translational Medical Science, Section of Pediatrics University of Naples “Federico II” Naples Italy; ^6^ Unit of Pediatric Gastroenterology and Endoscopy, Department of Pediatrics “S. Spirito” Hospital of Pescara Pescara Italy; ^7^ Pediatric Gastroenterology Unit Maggiore Hospital Bologna Italy; ^8^ Department of Pediatrics, Vittore Buzzi Children's Hospital University of Milan Milan Italy; ^9^ Division of Pediatrics, Department of Maternal and Child Health ASST Grande Ospedale Metropolitano Niguarda Milan Italy; ^10^ Department of Neurofarba, Meyer Children's Hospital University of Florence Florence Italy; ^11^ Pediatrics – IRCCS Casa Sollievo della Sofferenza San Giovanni Rotondo” Foggia Italy; ^12^ Unit of Gastroenterology, Digestive Endoscopy, Hepatology and Care of the Child with Liver Transplantation University Hospital of Padova Padova Italy; ^13^ Institute for Maternal and Child Health IRCCS “Burlo Garofolo” Trieste Italy; ^14^ Pediatrics, Fondazione IRCCS San Gerardo dei Tintori Monza Italy; ^15^ Paediatric Hepatology, Gastroenterology and Transplantation, ASST Papa Giovanni XXIII Bergamo Italy; ^16^ Pediatric Gastroenterology, Hepatology and Cystic Fibrosis Unit, Department of Pathophysiology and Transplantation Università degli Studi di Milano, Fondazione IRCCS Cà Granda, Ospedale Maggiore Policlinico di Milano Milan Italy

**Keywords:** anemia of chronic disease, epidemiology, iron deficiency anemia

## Abstract

**Objectives:**

We determined the prevalence of anemia and its characteristics in children with newly diagnosed inflammatory bowel disease (IBD) and investigated its trend during follow‐up.

**Methods:**

An observational, multicenter cohort study of IBD children with anemia at the diagnosis enrolled in the Italian Society of Pediatric Gastroenterology, Hepatology, and Nutrition IBD registry. Data were collected at the diagnosis and at 1 year.

**Results:**

Five hundred eighty‐nine (295 Crohn's disease [CD] [50%] and 294 ulcerative colitis [UC]/IBD unclassified [IBDU] [50%]) of 1634 patients with IBD presented with anemia (36%). Anemia rate was higher in CD than in UC (39% vs. 33%, *p* = 0.009), and most patients had moderate anemia (55%). Children with CD had higher rates of mild anemia than UC (38% vs. 33%, *p* < 0.0001), while severe anemia was more common in UC (13% vs. 6%, *p* = 0.001). In CD, lower age at the diagnosis and lower albumin level correlated with anemia severity (*p* = 0.0007 and <0.0001, respectively). In UC, severe disease was more common in patients with severe anemia compared to those with mild and moderate anemia (20.6% vs. 43.6%, *p* = 0.01; 17% vs. 43.6%, *p* = 0.001). At 1 year, 99 children (22.9%) were persistently anemic and were characterized by a more severe disease compared to those who had resolved their anemia.

**Conclusions:**

More than one third of IBD children present with anemia, most commonly moderate. Severe anemia is more common in UC compared to CD. One in four patients is still anemic after 1 year from the diagnosis, suggesting inadequate attention to the issue and the need for dedicated therapeutic management and careful monitoring.

## Abstract (highlights)

**What is Known:**

- Anemia, the most common extraintestinal manifestation of inflammatory bowel disease (IBD), has a multifactorial etiology and significantly impacts patients' quality of life

**What is New:**

- Anemia at diagnosis was less prevalent than previously reported, affecting approximately one third of the patients.
- Iron deficiency was the most common type of anemia in children with IBD, while anemia of chronic disease was more frequent in Crohn's disease compared to ulcerative colitis.
- Standard IBD medical care led to a 23% persistence rate, emphasizing the importance of dedicated therapeutic management, including monitoring and adequately treating iron deficiency to prevent recurrence alongside addressing underlying intestinal inflammation.

## Graphical abstract

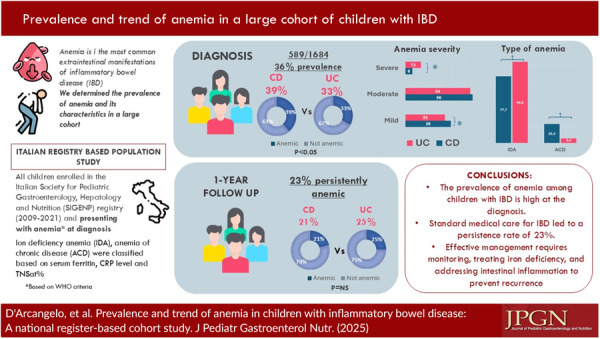

## 1 INTRODUCTION

Anemia is among the most common extraintestinal manifestations of inflammatory bowel disease (IBD)^1^, ^2^, ^3^, ^4^ and impacts patients' quality of life.^5^, ^6^ Its etiology is multifactorial; in both Crohn's disease (CD) and ulcerative colitis (UC) a combination of factors, including reduced food intake, malabsorption due to gastrointestinal inflammation, chronic inflammation, and blood loss, can cause different types of anemia.^2^ In 2020, the North American Society for Pediatric Gastroenterology, Hepatology, and Nutrition IBD committee published a position paper^2^ aligning with the European Crohn's and Colitis Organization (ECCO) guidelines for adults with IBD.^1^ Both recommend initial screening tests for anemia at diagnosis, with repeated screenings every 3 months for patients with active disease and every 6–12 months for those with inactive disease. Recently, an ECCO update on IBD and anemia was published, reaffirming the recommendation for regular assessment in patients due to its high prevalence and impact on quality of life.^3^ Dedicated Italian pediatric guidelines also confirm the importance of the topic.^7^ The prevalence of anemia in the pediatric setting ranges from 44% and 74% at diagnosis and 25% and 58% after 1 year follow‐up,^4^, ^8^, ^9^, ^10^, ^11^ being higher in children than in adolescents.^12^ This heterogeneity reflects the lack of clear recommendations for screening, testing, and treating this condition. Additionally, the small sample sizes and retrospective nature of most of these studies limit their findings. In adults, the results of a multicenter, observational, longitudinal, prospective study on the prevalence, pathogenesis, and treatment of anemia in IBD were recently published by the Italian Group for IBD: anemia was detected in 737 out of 5416 new‐diagnosed patients (13.6% prevalence).^13^ To better understand the prevalence and characteristics of anemia in children with IBD, we performed a comprehensive register‐based population study. Our primary aim was to evaluate the prevalence of anemia and its characteristics in children with newly diagnosed IBD. The secondary objectives were to define predictive factors for the severity of anemia and its persistence at a 1‐year follow‐up.

## 2 METHODS

### 2.1 Study design

We performed a retrospective observational nationwide register‐based cohort study exploring the prevalence of anemia at the diagnosis in children with IBD. The study population included all children prospectively enrolled in the Italian Society for Pediatric Gastroenterology, Hepatology, and Nutrition (SIGENP) registry who presented with anemia upon diagnosis. The registry started on January 1, 2009, and included patients less than 18 years old with a new diagnosis of IBD. Data of all anemic children enrolled and stored in the registry from January 1, 2009, to April 22, 2021 (the data retrieval date) were used for this study. The methodology of the registry has been previously described in detail.^14^

### 2.2 Data collection and anemia definition

We collected data from all patients under 18 years of age who were newly diagnosed with IBD and anemia. The following mandatory laboratory data were available at the time of inclusion: hemoglobin (Hgb, g/dL), mean corpuscular volume (MCV, fL), ferritin (μg/L), C‐reactive protein (CRP), and/or transferrin saturation (TNSat, %). Patients with incomplete laboratory data for the definition of anemia at the diagnosis were excluded. The diagnosis of IBD was based on clinical history, physical examination, endoscopic, histologic, and imaging findings, as for the Porto criteria for the diagnosis of IBD^15^ Both CD and UC patients were included. Data collected for this study included demographic data (date of birth, gender, age at the diagnosis), auxological parameters (weight [kg], height [cm], body mass index [BMI] [kg/m^2^] with respective standard deviations [SDs]), disease activity indexes (including weighted Pediatric Crohn's disease activity index [wPCDAI]^16^ for CD and pediatric ulcerative colitis activity index [PUCAI] for UC),^17^ disease location and behavior according to the Paris classification.^18^ The presence of anemia at diagnosis was defined based on the World Health Organization (WHO) criteria.^19^, ^20^ Specifically, in children under 5 years old, anemia was defined by an Hgb concentration below 11 g/dL, in those aged between 5 and 11 years by an Hgb below 11.5 g/dL, while individuals aged between 12 and 14 years were considered anemic if their Hgb was below 12 g/dL; for nonpregnant girls over 15 years old, anemia was identified by a Hgb level under 12 g/dL, and for boys over 15 years old, anemia was defined as a Hgb concentration below 13 g/dL.

The grading of anemia into mild, moderate, and severe was based on the same WHO criteria as follows: mild anemia was defined by Hgb 10–10.9 g/dL in children under 5 years old, 11–11.9 g/dL in children 5–14 and in nonpregnant girls >15 years, and by Hgb 11–12.9 g/dL in boys >15 years; anemia was defined as moderate by a Hgb concentration ranging from 7 to 9.9 g/dL in children <5 years of age, and from 8 to 10.9 g/dL in all the other age groups; anemia was deemed as severe when Hgb concentrations were lower than 8 g/dL in all the age group except in those children <5 years were the Hgb concentration for severe anemia needed to be <7 g/dL.

Microcytic anemia was defined as an MCV <80 fL, normocytic anemia as an MCV ranging from 80 to 99 fL, and macrocytic by an MCV >99 fL.

When available, serum ferritin and TNSat% were used to define anemia subtypes as follows: iron deficiency anemia (IDA) was diagnosed when serum ferritin levels were below 30 μg/L, and TNSat was less than 16%. In the presence of active biochemical inflammation (with CRP levels exceeding 10 mg/L), serum ferritin greater than 100 μg/L and TNSat less than 20% defined anemia of chronic disease (ACD), while children with ferritin levels between 30 and 100 μg/L and a TNSat below 16% were classified as having iron deficiency.^3^ Data on treatment received (including exclusive enteral nutrition, mesalamine, sulfasalazine, anti‐TNF‐α [infliximab and adalimumab], immunomodulators [azathioprine and methotrexate]) at the diagnosis were extracted as well.

At 1‐year follow‐up, laboratory parameters (Hgb, MCV, ferritin, serum iron, TNSat, and CRP) were collected to determine the persistence of anemia.

### 2.3 Statistical analysis

All data were summarized and displayed as mean ± SD for the normally distributed continuous variables. Non‐normally distributed variables were presented as medians (interquartile ranges). Categorical data were expressed as frequencies and percentages. Comparison of groups was performed using Student's *t* test for unpaired data in a two‐group comparison. One‐way analysis of variance followed by the Bonferroni post hoc test was used for multiple group comparisons. Chi‐square tests with Fisher's correction were used to address any differences for categorical variables, as needed. For multiple group comparisons, a post hoc Bonferroni correction was applied for significance (alphaBonf = 0.05/3 = 0.0167). The Pearson correlation coefficient was used to assess the correlation between the Hgb values and the biological (albumin, ESR, and CRP) and clinical (wPCDAI and PUCAI) disease severity index. Univariate and multivariate logistic stepwise regression analyses were used to analyze the association between the persistence of anemia at 1‐year follow‐up and the characteristics of IBD patients. The following covariates were selected a priori (based on clinical significance) for inclusion in multivariable analyses: demographics (including age and gender and auxological parameters), main laboratory parameters at the diagnosis reflecting disease activity (ESR, CRP, albumin, and Hgb), clinical and endoscopic disease activity indexes (wPCDAI/PUCAI, SES‐CD, and UCEIS, respectively for CD and UC), upfront anti‐TNF therapy for CD, induction of remission with systemic corticosteroids and acute severe colitis at the diagnosis for UC. In the final model, the dependent variable was the persistence of anemia at 1‐year follow‐up, and the covariates were those with significance ≤0.1 in the univariate analysis. A *p* value of 0.05 or less was considered as significant.

### 2.4 Ethics

The study was conducted in accordance with the Declaration of Helsinki and was consistent with good clinical practice. The registry study protocol was reviewed and approved by the Umberto I Hospital Independent Ethics Committee. Patients or their legally acceptable representatives provided written informed consent.

## 3 RESULTS

### 3.1 Primary outcome evaluation: Prevalence of anemia

During the inclusion period, 2780 children diagnosed with IBD were enrolled in the registry (1276 CD and 1504 UC). Of these, 1146 were excluded due to a lack of mandatory laboratory values at diagnosis. Thus, 1634 patients were enrolled in this study. Out of the 1634 patients included (748 CD and 886 UC), 589 were diagnosed with anemia (295 with CD and 294 with UC), resulting in a prevalence of 36%. Anemia was significantly higher in CD than in UC (39% vs. 33%, *p* = 0.009).

### 3.2 General features of study population and characteristics of anemia

The baseline demographic and clinical characteristics of the entire population and the differences between UC and CD patients are reported in Table 1.

**Table 1:** Baseline demographic characteristics of the population.

	Total anemic IBD patients (*n* = 589)	Anemic CD patient (*n* = 295)	Anemic UC patient (*n* = 294)	*p*
Age at diagnosis, median (IQR)	12.3 (8.7–14.15)	12.8 (10.2–14.58)	11.1 (6.9–13.8)	<0.0001
Female sex, *n* (%)	279 (47.5)	114 (39)	165 (52)	<0.0001
PUCAI, median (IQR)			35 (25–50)	
wPCDAI, median (IQR)		20 (10–33)		
SES‐CD, median (IQR)		10.5 (5–17)		
Mayo, median (IQR)			4 (3–6)	
UCEIS, median (IQR)			6 (5–7)	
CRP (mg/L), median (IQR)	2.1 (0.33–7)	3.42 (1–12.92)	0.6 (0.2–3.3)	0.03
ESR (mm/h), median (IQR)	43 (25–67.5)	51 (31.5–76)	32 (16–54.2)	0.0001
Albumin (g/L), median (IQR)	35 (31–41)	34.7 (30–39)	40 (34–43)	<0.0001
CD location, *N* (%)				
L1		50 (17)		
L2		54 (18)		
L3		181 (61)		
L4a		49 (17)		
L4b		24 (8)		
L4ab		6 (2)		
p		14 (5)		
Behavior, *N* (%)				
B1		234 (79)		
B2		56 (19)		
B3		5 (2)		
G1		132 (45)		
UC location, *N* (%)				
E1			19 (6.5)	
E2			53 (18)	
E3			55 (19)	
E4			167 (57)	
S1			64 (22)	

Abbreviations: CD, Crohn's disease; CRP, C‐reactive protein; ESR, erythrocyte sedimentation rate; IBD, inflammatory bowel disease; IQR, interquartile range; PUCAI, pediatric ulcerative colitis activity index; SD, standard deviation; SES‐CD, Simple Endoscopic Score for Crohn's disease; UC, ulcerative colitis; UCEIS, ulcerative colitis endoscopic index of severity; wPCDAI, weighted pediatric Crohn's disease activity index.

Table 2 shows anemia data for pediatric IBD patients and compares anemia characteristics between CD and UC.

**Table 2:** Baseline anemia characteristics and difference between CD and UC children.

	Anemic IBD patient (*n* = 589)	Anemic CD patient (*n* = 295)	Anemic UC patient (*n* = 294)	*p*
Hgb (g/L), median (IQR)	10.5 (9.5–11.2)	10.5 (9.4–11)	10.7 (9.3–11.53)	0.19
MCV (fL), mean ± SD	73 ± 7.7	71.7 ± 7.3	74 ± 7.9	0.0003
Ferritin (μg/L), median (IQR)	20 (8.2–69.5)	58 (25–103)	12 (7–20)	0.0005
Serum iron (μmol/L), median (IQR)	14 (4.3–24.68)	14 (4.3–22)	12.5 (4.5–24.68)	0.11
TSat (%), median (IQR)	10.6 (6–15)	10.3 (7.5–13.4)	10.8 (6–16)	0.36
Anemia severity, *n* (%)				
Mild	209 (35.5)	112 (38)	97 (33)	<0.0001
Moderate	324 (55)	166 (56.3)	158 (53.8)	0.6
Severe	56 (9.5)	17 (5.7)	39 (13.2)	0.0019
Type of anemia, *n* (%)				
Microcytic	478 (81)	257 (87)	221 (75)	0.0002
Normocytic	111 (19)	38 (13)	73 (25)	
Anemia subtypes, *n* (%)^a^				
IDA	471 (87.7)	202 (77.7)	262 (94.6)	<0.0001
ACD	66 (12.3)	58 (22.3)	15 (5.4)	

Abbreviations: ACD, anemia of chronic disease; CD, Crohn's disease; Hgb, hemoglobin; IBD, inflammatory bowel disease; IDA, iron deficiency anemia; IQR, interquartile range; MCV, mean corpuscular volume; SD, standard deviation; TSat, transferrin saturation; UC, ulcerative colitis.

^a^ Fifty‐two patients were excluded from this analysis.

Most children presented with moderate (55%) and microcytic anemia (81%). IDA was diagnosed in 471/537 (87.7%) of children with available data of TNsat%. Sixty‐six (12.3%) were classified as ACD. Fifty‐two children could not be labeled without data on TNSat% and/or CRP. A higher proportion of children with CD showed mild anemia compared to UC (38% vs. 33%, *p* < 0.0001), while severe anemia was more common in UC than in CD (13.2% vs. 5.7%, *p* = 0.0019).

Patients with UC showed a higher frequency of IDA compared to CD (94.6% vs. 77.7%), whereas CD patients had a higher frequency of ACD compared to UC patients (22.3% vs. 5.4%, *p* < 0.0001).

### 3.3 Characteristics according to anemia severity in children with CD

Table 3 analyzes and compares the characteristics of patients with CD based on the severity of anemia at diagnosis.

**Table 3:** Characteristics of patients with CD and UC according to the severity of anemia.

CD	Mild anemia (*n* = 112)	Moderate anemia (*n* = 166)	Severe anemia (*n* = 17)	*p* *
Age at diagnosis, median (IQR)	13.25 (11.35–15.3)	12.25 (9.5–13.8)	11.45 (3.77–13.23)	0.0007
				0.0008,^a^ 0.1,^b^ 0.4^c^
Sex F, *N* (%)	36 (32)	70 (42)	8 (47)	0.15
wPCDAI, median (IQR)	18 (5–33)	22.25 (13–33)	20 (10–40.63)	0.15
SES‐CD, median (IQR)	11 (5.5–16.5)	12 (5–20)	8 (4–15)	0.39
CRP (mg/dL), median (IQR)	2.84 (0.83–8.26)	3.67 (1.1–20.9)	12.61 (2.47–68.45)	0.37
ESR (mm/h), median (IQR)	54 (32–78)	52.5 (32.25–78)	43.5 (30.5–95)	0.96
Albumin (g/L), median (IQR)	35 (32.75–40.25)	33.35 (29.4–38)	24 (21.5–29)	<0.0001
				0.03,^a^ <0.0001,^b^ 0.0005^c^
CD location, *N* (%)				
L1	23 (20.5)	25 (15)	2 (12)	0.41
L2	22 (19.6)	27 (16)	5 (29.4)	
L3	61 (54.5)	111 (67)	9 (53)	
Upper GI involvement, *N* (%)	79 (29.4)	37 (22)	3 (17.6)	0.3
Behavior, *N* (%)				
B1	89 (79.5)	130 (78)	15 (88)	0.71
B2	20 (18)	34 (20.5)	2 (12)	
B3	3 (3)	2 (1)	0	
G0	56 (50)	98 (59)	9 (53)	0.32

UC	Mild anemia (*n* = 97)	Moderate anemia (*n* = 158)	Severe anemia (*n* = 39)	*p* *
Age at diagnosis, median (IQR)	12.45 (5.4–4.7)	10.9 (7–13.7)	12.3 (7.5–14.3)	0.86
Sex F, *N* (%)	46 (47.4)	92 (58.2)	27 (69.2)	0.05
PUCAI, median (IQR)	35 (25–50)	35 (20–45)	40 (26.2–58.7)	0.04
				1,^a^ 0.2,^b^ 0.04^c^
Mayo, median (IQR)	5 (3–6)	4 (2–5)	4 (3–5)	0.09
UCEIS, median (IQR)	6 (5–7)	6 (5–7)	6 (6–7)	0.8
CRP (mg/dL), median (IQR)	0.5 (0.15–5.33)	0.7 (0.2–3)	2.73 (0.29–7.9)	0.09
ESR (mm/h), median (IQR)	31 (18–51)	33.5 (16.2–55.7)	45 (23.5–73)	0.07
Albumin (g/L), median (IQR)	38.5 (31.8–42.25)	39.4 (33–43.6)	35 (32.5–37.2)	0.63
UC location, *N* (%)				
E1	9 (9.3)	8 (5)	2 (5)	0.19
E2	21 (21.6)	26 (16.4)	6 (15.4)	
E3	10 (10.3)	35 (22)	10 (25.6)	
E4	57 (58.8)	89 (56.3)	21 (54)	
S1	20 (20.6)	27 (17)	17 (43.6)	0.0015
				0.5,^a^ 0.01,^b^ 0.001^c^

Abbreviations: ANOVA, analysis of variance; CD, Crohn's disease; CRP, C‐reactive protein; ESR, erythrocyte sedimentation rate; F, female; IQR, interquartile range; N, number; PUCAI, pediatric ulcerative colitis activity index; SD, standard deviation; SES‐CD, Simple Endoscopic Score for Crohn's disease; UCEIS, ulcerative colitis endoscopic index of severity; wPCDAI, weighted pediatric Crohn's disease activity index.

* Adjusted *p* < 0.05, after post hoc Bonferroni test for continuous variable; for binomial variables alphaBonf = 0.05/3 = 0.0167.

^a^ Mild vs. moderate.

^b^ Mild vs. severe.

^c^ Moderate vs. severe were presented only if *p* < 0.05 for ANOVA and *p* < 0.01 for the chi‐square test.

Patients with CD and moderate anemia experienced symptoms at an earlier age, compared to those with mild (12.25 [9.5–13.8] vs. 13.25 [11.35–15.3] years, *p* = 0.0008).

Additionally, lower levels of albumin at the time of diagnosis are linked to the severity of anemia. Patients with severe anemia had significantly lower values compared to those with mild and moderate anemia (24 [21.5‐29] vs. 35 [32.7–40.2], *p* < 0.0001; 24 [21.5–29] vs. 33.3 [29.4–38], *p* = 0.0005).

A weak, although significant, correlation was observed between Hgb levels and wPCDAI (*r* = −0.29, *p* < 0.0001), albumin (*r* = 0.35, *p* < 0.0001), ESR (*r* = −0.20, *p* = 0.0007), auxological parameters (weight SD [*r* = 0.18, *p* = 0.003], height SD [*r* = 0.23, *p* = 0.0004]). No significant correlation was found between Hgb levels and SES‐CD, CRP, and BMI SD (data not shown).

### 3.4 Characteristics according to anemia severity in children with UC

Table 3 presents an analysis and comparison of the characteristics of patients diagnosed with UC, categorized by the severity of anemia at diagnosis.

Severe disease (S1) was more common in patients with severe anemia than in those with mild and moderate anemia (20.6% vs. 43.6%, *p* = 0.01; 17% vs. 43.6%, *p* = 0.001). The PUCAI score significantly differed between the three groups (*p* = 0.04): patients with severe anemia had a higher PUCAI than those with moderate anemia (40 [26.2–58.75] vs. 35 [20–45], *p* = 0.04).

A significant, although weak, correlation was observed between Hgb levels and PUCAI (*r* = −0.27, *p* = 0.01), while no significant correlations were observed with other biological and auxological parameters (data not shown).

### 3.5 Persistence of anemia at 12‐month follow‐up and analysis of associated factors

One hundred and fifty‐eight children did not have complete laboratory data for the definition of anemia at 1‐year follow‐up and were excluded for subsequent analysis. Ninety‐nine out of 431 patients (22.9%) were still anemic after 1 year. Specifically, 46 children with CD out of 222 (20.7%) and 53 children with UC out of 209 (25.3%) were anemic, with no statistically significant difference between the two groups (*p* = 0.3).

When evaluating the characteristics of patients with persistent anemia after 12 months of diagnosis (Supporting Information S1: Table 1), there were no significant differences between persistently anemic patients with CD and UC in terms of anemia characteristics. The analysis and comparison of the demographics, IBD characteristics, and treatment received are reported in Table 4. Information on the type of iron treatment (oral or intravenous [IV]) was available for 91 children with CD (31%) and 73 children with UC (25%). Among them, 22 children with CD (24%) and 19 with UC (26%) received IV iron supplementation at diagnosis (*p* = 0.85). No significant difference was observed in the type of iron supplementation between children with and without persistent anemia at 1 year (data not shown).

**Table 4:** Demographic and IBD characteristics in patients with and without persistence of anemia at 12‐month follow‐up.

CD	Anemic at 1 year (*n* = 46)	Not anemic at 1 year (*n* = 176)	*p*
Age at diagnosis, median (IQR)	12.8 (9.3–14.3)	12.6 (9.9–14.2)	0.7
Sex F, *N* (%)	17 (37)	69 (39)	0.8
wPCDAI at T0, median (IQR)	17.5 (13–33)	20 (10–33)	0.5
wPCDAI at T1, median (IQR)	5 (0–22.5)	0 (0–5)	0.47
SES‐CD at T0, median (IQR)	11 (4–16)	11 (5–18)	0.3
CRP (mg/dL) at T0, median (IQR)	3.3 (1.3–20)	3.3 (1–11)	0.16
CRP (mg/dL) at T1, median (IQR)	0.2 (0–0.5)	0.3 (0.1–1.1)	0.48
ESR (mm/h) at T0, median (IQR)	48.5 (34.2–82.5)	53 (32–76)	0.6
ESR (mm/h) at T1, median (IQR)	25 (2–47)	17 (8–28.5)	<0.0001
Albumin (g/L) at T0, median (IQR)	35 (29–40)	34 (30–38)	0.6
Albumin (g/L) at T1, median (IQR)	38 (28–42)	43 (38.1–45)	0.48
Hgb (g/dL) at T0, median (IQR)	10.2 (9.6–11.3)	10.7 (9.8–11.4)	0.8
MCV (fL) at T0, median (IQR)	69 (64–75.5)	72 (67–77)	0.2
CD location at T0, *N* (%)			
L1	11 (24)	35 (20)	0.5
L2	11 (24)	31 (17.6)	0.3
L3	24 (52)	95 (54)	0.8
L4a	4 (9)	28 (16)	0.2
L4b	2 (4)	29 (16.4)	0.03
*p*	7 (15)	26 (15)	1
Behavior at T0, *N* (%)			
B1	34 (74)	151 (86)	0.07
B2	9 (20)	22 (12)	0.2
B3	3 (6.5)	4 (2)	0.1
G1	13 (28)	46 (26)	0.8
CD treatment at the diagnosis			
5‐ASA	11 (24)	22 (12.5)	0.06
IM	21 (45.5)	63 (36)	0.2
Anti‐TNFα	13 (28)	72 (41)	0.1
EEN	22 (48)	97 (55)	0.4
Steroids	10 (22)	52 (29.5)	0.3
PEN+Diet	0	16 (9)	0.04
Surgery	0	2 (1.3)	1
CD treatment at the 1 year			
IM	14 (30)	76 (43)	0.09
Anti‐TNFα	21 (46)	78 (44)	0.8
Ustekinumab	1 (2)	4 (2)	1
PEN+Diet	9 (19.5)	21 (12)	0.2
Steroids	4 (9)	17 (10)	1
Surgery	1 (2)	6 (3)	

UC	Anemic at 1 year (*n* = 53)	Not anemic at 1 year (*n* = 156)	*p*
Age at diagnosis, median (IQR)	12 (8.9–13.5)	10.8 (6.8–14)	0.3
Sex F, *N* (%)	31 (58.5)	87 (56)	0.7
PUCAI at T0, median (IQR)	32.5 (20–51.2)	35 (25–50)	0.9
PUCAI at T1, median (IQR)	20 (0–36.25)	0 (0–20)	0.0008
UCEIS at T0, median (IQR)	6 (5–6)	6 (5–7)	0.6
CRP (mg/dL) at T0, median (IQR)	1.2 (0.3–3.7)	0.5 (0.1–4)	0.4
CRP (mg/dL) at T1, median (IQR)	0.8 (0.2–6)	0.2 (0.07–0.69)	0.9
ESR (mm/h) at T0, median (IQR)	34 (22–52)	32 (18–59)	0.6
ESR (mm/h) at T1, median (IQR)	25 (13.5–57.2)	14.5 (6–24.5)	0.19
Albumin (g/L) at T0, median (IQR)	36 (32–41)	38 (33–43)	0.7
Albumin (g/L) at T1, median (IQR)	38 (35–43)	42 (38.5–45)	0.4
Hgb (g/dL) at T0, median (IQR)	10.1 (8.4–10.9)	9.9 (8.4–10.9)	0.7
MCV (fL) at T0, median (IQR)	71 (66–80)	74 (68–80)	0.5
UC location at T0, *N* (%)			
E1	2 (4)	11 (7)	0.5
E2	10 (19)	28 (18)	0.8
E3	10 (9)	24 (15)	0.5
E4	31 (58.5)	93 (59.5)	1
S1 at T0	16 (30)	24 (15)	0.02
UC treatment at the diagnosis			
5‐ASA	33 (62)	131(84)	0.001
IM	19 (36)	38 (24)	0.1
Anti‐TNFα	9 (17)	25 (16)	0.8
Steroids	23 (43)	78 (44)	0.4
Colectomy	0	2 (1)	1
UC treatment at the 1 year			
5‐ASA	20 (38)	95 (61)	0.004
IM	21 (40)	62 (40)	1
Anti‐TNFα	19 (36)	60 (38)	0.8
Vedolizumab	1 (2)	3 (2)	1
Steroids	23 (43)	22 (14)	<0.0001
Colectomy	2 (4)	4 (2.6)	0.6

Abbreviations: 5‐ASA, 5‐aminosalicylic acid; Anti‐TNFα, anti‐tumor necrosis factor alpha; CD, Crohn's disease; CRP, C‐reactive protein; EEN, exclusive enteral nutrition; ESR, erythrocyte sedimentation rate; F, female; Hgb, hemoglobin; IBD, inflammatory bowel disease; IM, immunomodulators; IQR, interquartile range; MCV, mean corpuscolar volume; N, number; PEN, partial enteral nutrition; PUCAI, pediatric ulcerative colitis activity index; SD, standard deviation; SES‐CD, Simple Endoscopic Score for Crohn's disease; UC, ulcerative colitis; UCEIS, ulcerative colitis endoscopic index of severity; wPCDAI, weighted pediatric Crohn's disease activity index.

Factors predicting anemia persistence at 12 months were analyzed using univariate and multivariate logistic regression analysis (Supporting Information S2: Table 2). In CD, independent predictors for anemia persistence were the wPCDAI (*p* = 0.0005, odds ratio [OR]: 0.7, 95% confidence interval [CI]: 0.6–0.8) and SES‐CD (*p* = 0.0009, OR: 1.3, 95% CI: 1.1–1.5). No variable was identified as an independent predictor of anemia persistence in UC.

## 4 DISCUSSION

Anemia is one of the most common extraintestinal manifestations in children with IBD, therefore fully understanding and characterizing it in this population is crucial. To the best of our knowledge, our study reports the largest cohort of anemic children with IBD at the diagnosis published so far.

We found that more than one third of children newly diagnosed with IBD have anemia. Smaller retrospective series generally reported a higher prevalence rate, around 70%,^10^, ^21^, ^22^ However, in line with our results, a large prospective cohort study—the ICURE study, which included 749 patients (children and adults) with IBD—reported a 30% prevalence of anemia at diagnosis, with a higher prevalence among children (54%) than adults (27%).^23^ A prevalence similar to ours was found by Carvalho et al. in their retrospective cohort of 69 patients, reporting an IDA prevalence of 43.5%.^24^ This is also one of the most recent studies to investigate the issue of anemia, suggesting a more accurate definition of the condition and possibly reflecting a reduction in the diagnostic delay over time. A similar observation is reported in a meta‐analysis of data exclusively from adult IBD cohorts, which found an overall anemia prevalence of 24% in IBD patients across European countries, with a prevalence of 37% reported in Italy.^4^

At the diagnosis, the higher prevalence of anemia in CD compared to UC (39% vs. 33%), as emerged from our data, is common across other studies.^9^, ^23^, ^25^ This may be the result of multiple elements: on one hand, children with CD, particularly those with upper gastrointestinal involvement, may have poor iron absorption, but, the higher prevalence of ACD in CD compared to UC could reflect the significant impact of all the complex inflammation‐related factors such as lower albumin levels, hepcidin‐mediated decreased iron absorption, reduced production of erythropoietin and decreased density of its receptors, enhanced erythrophagocytosis, and premature apoptosis of erythroid precursor cells.^2^

At 1‐year follow‐up, 22.9% of children still had anemia despite receiving medical treatment for IBD (data recorded and analyzed only at diagnosis, with no information on subsequent therapeutic management). These patients showed signs of more severe disease, as indicated by higher PUCAI scores in UC and higher ESR levels in CD. Although our prevalence data at diagnosis is lower than that reported in the literature,^21^, ^22^, ^26^ our persistence data at follow‐up is more consistent with other reports, which show anemia persistence rates of 17%–20%^9^, ^23^ and suggest that this rate stabilizes over a 10‐year follow‐up period. In the absence of a positive trend in terms of outcomes, we must note with disappointment that despite advancements in therapeutic options for IBD, these have not significantly impacted the prevalence of anemia at follow‐up. Our data also indicate that the impact of ACD decreases over time, suggesting that untreated anemia and iron deficiency are the primary reasons for its persistence. This underscores the importance of ongoing monitoring even after anemia correction, as emphasized by the ECCO and Italian SIGENP guidelines.^3^, ^7^ Experts recommend focusing on correcting Hgb levels and replenishing iron stores adequately to prevent anemia recurrence, with a proactive approach to maintain ferritin levels above 100 ng/mL.^3^ This is particularly crucial given the growing body of literature highlighting the importance of addressing non‐anemic iron deficiency: this condition is a precursor to anemia and is linked to symptoms such as reduced physical performance, cognitive impairment, fatigue, headache, sleep disorders, and restless legs syndrome,^3^, ^27^, ^28^ warranting appropriate management as recommended by the latest ECCO guidelines on EIMs.^3^ A comprehensive analysis of the impact of specific anemia treatments was beyond the scope of this study due to a lack of data on treatment type, dosage, and duration for many patients. However, we analyzed the IBD‐related treatment both at the diagnosis and at 1‐year follow‐up, as well as its association with anemia persistence. We did not find any significant associations, except for potential indicators of a milder onset in UC patients who resolved their anemia, reflected by the higher rate of 5‐aminosalicylic acid treatment received at the diagnosis and the reduced need for steroids at 1‐year follow‐up. Our study has some limitations, mainly due to incomplete data on transferrin and transferrin saturation, as well as the absence of data on soluble transferrin receptors. These parameters would have provided additional insights into the relative contributions of iron deficiency and inflammation to the pathogenesis of anemia. Additionally, many patients were excluded due to incomplete data at diagnosis, potentially affecting the comprehensiveness of the findings. Finally, other limitations include the lack of information on the therapeutic management of anemia which prevented us from investigating its effect on persistence, as well as the absence of a control group of non‐anemic children, which could have strengthened our observations, confined to anemic IBD patients. Despite these limitations, our study has significant strengths. First, we examined a large sample size (the largest in pediatrics), which adds to the reliability of our results. Moreover, we used a strict definition of anemia according to WHO guidelines, ensuring consistency with other data in the field. In conclusion, our study confirmed a significant prevalence of anemia among children with IBD, both at the diagnosis and 1 year later. Despite recommendations and increased awareness raised by organizations such as ECCO, there remains insufficient attention to and therapeutic management of this issue. This is particularly critical in children, as unrecognized and untreated iron deficiency can significantly impact their development and cognitive performance.

## CONFLICT OF INTEREST STATEMENT

The authors declare no conflicts of interest.

## Supporting information

Supporting information.

Supporting information.
