# Supplementary material for: Prevalence and trend of anemia in children with inflammatory bowel disease: A national register‐based cohort study
Source: J Pediatr Gastroenterol Nutr. 2025 Mar 31;80(6):967–78. doi: 10.1002/jpn3.70029 (PMC12133643; doi:10.1002/jpn3.70029)
Supplement: Supplementary file 1 — Supporting information. [file JPN3-80-967-s002.docx]

| *Supplementary Table1. Anemia characteristics in patients with persistence of anemia at 12 months follow-up* | | | | |
| --- | --- | --- | --- | --- |
|  | **Anemic IBD patients (n=99)** | **Anemic CD patients (n=46)** | **Anemic UC patients (n=53)** | **p** |
| **Hgb (g/L), median (IQR)** | 10.2 (9.6-11.3) | 10.4 (9.7-11.4) | 10.1(8.6-10.9) | 0.27 |
| **MCV (fL), median (IQR)** | 71 (64-77) | 69 (64-75.5) | 71 (66-80) | 0.25 |
| **Ferritin (μg/L), median (IQR)** | 17 (7-43) | 38.5 (14.5-124) | 9.3 (5.5-18.9) | 0.84 |
| **Serum Iron (μg/dL), median (IQR)** | 16.5 (4-28) | 14.4 (4.2-27.7) | 19 (4.1-37.6) | 0.43 |
| **TSat (%), median (IQR)** | 9.7 (6-14.5) | 9.4 (5.5-15) | 10.2 (7-13) | 0.4 |
| **Anemia severity, n (%)**  Mild  Moderate  Severe | 52 (52.5)  43 (43.4)  4 (4.1) | 26 (56.6)  18 (39.1)  2 (4.3) | 26 (49.1)  25 (47.2)  2 (3.7) | 1  0.54  1 |
| **Type of anemia, n (%)**  Microcytic  Normocytic | 75 (75.8)  24 (24.2) | 32 (69.6)  14 (30.4) | 43 (81.1)  10 (18.9) | 0.2 |
| **Anemia subtypes, n (%)***  IDA  ACD | 75 (91.5)  7 (8.5) | 36 (87.8)  5 (12.2) | 39 (95)  2 (5) | 0.43 |
| *IBD: inflammatory bowel disease; CD: Crohn’s disease; UC: ulcerative colitis; IQR: interquartile range; Hgb: hemoglobin; MCV: mean corpuscular volume; TSat: transferrin saturation; IDA: iron deficiency anemia; ACD: anemia of chronic disease. *17 patients excluded from this analysis.* | | | | |
