# Supplementary material for: Prevalence and trend of anemia in children with inflammatory bowel disease: A national register‐based cohort study
Source: J Pediatr Gastroenterol Nutr. 2025 Mar 31;80(6):967–78. doi: 10.1002/jpn3.70029 (PMC12133643; doi:10.1002/jpn3.70029)
Supplement: Supplementary file 2 — Supporting information. [file JPN3-80-967-s001.docx]

| *Supplementary Table 2. Univariate and multivariate logistic regression analysis of factors associated with the persistence of anemia in CD and UC* | | | | | | |
| --- | --- | --- | --- | --- | --- | --- |
| **CD** | **Univariate analysis** | **Multivariate analysis** | | | | |
|  | **p** | **OR (95% CI)** | | **p** | | |
| **wPCDAI**  **Age**  **Sex**  **SES-CD**  **Albumin (g/L)**  **ESR (mm/h)**  **CRP (mg/L)**  **Hgb (g/L)**  **Upfront anti-TNF**  **Model characteristics**  AUC [95% CI] 0.78 [0.6-0.9]  R^2^ 0.4  PPV [%] 100  NPV [%] 89.8 | 0.01  0.69  0.39  0.04  0.17  0.68  0.57  0.73  0.08 | 0.7 (0.6-0.8)  1.3 (1.1-1.5)  2.6 (0.3-49.1) | | **0.0005**  **0.0009**  0.4 | | |
| **UC** | **Univariate analysis** | **Multivariate analysis** | | | | |
|  | **p** | **OR (95% CI)** | | **p** | | |
| **PUCAI**  **Age**  **Sex**  **UCEIS**  **ESR (mm/h)**  **Albumin (g/L)**  **S1**  **E4**  **Systemic CS induction** | 0.28  0.65  0.83  0.81  0.59  0.98  0.71  0.49  0.78 | - | | - | | |
| *CD: Crohn’s disease; UC: ulcerative colitis; OR: odds ratio; CI: confidence interval; wPCDAI: weighted pediatric Crohn’s disease activity index; SES-CD: Simple Endoscopic Score for Crohn’s disease; CRP: C-reactive protein; ESR: erythrocyte sedimentation rate; SD: standard deviation; PUCAI: pediatric ulcerative colitis activity index; UCEIS: ulcerative colitis endoscopic index of severity; CS: corticosteroids.* | | | | | | |
|  | | |  | |  |  |
